# The MHC (Major Histocmpatibility Complex) Exceptional Molecules of Birds and Their Relationship to Diseases

**DOI:** 10.3390/ijms26083767

**Published:** 2025-04-16

**Authors:** Antonio Arnaiz-Villena, Fabio Suarez-Trujillo, Valentin Ruiz-del-Valle, Ignacio Juarez, Christian Vaquero-Yuste, José Manuel Martin-Villa, Tomás Lledo

**Affiliations:** Department of Immunology, School of Medicine, Complutense University of Madrid, 28040 Madrid, Spain

**Keywords:** amino acid residues, *Aves*, beta-2-microglobulin, birds, *Carduelis*, codons, diseases, HLA, hydrogen bound, intron, malaria, Marek’s disease, MHC (Major Histocompatibility Complex), molecules, *Passeriformes*, *Serinus*, *Spinus*, vertebrates, wild birds

## Abstract

There are about 5000 species of *Passeriformes* birds, which are half of the extant ones. Their class I MHC molecules are found to be different from all other studied vertebrates, including other bird species; i.e., amino acid residues 10 and 96 are not the seven canonic residues extant in all other vertebrate molecules. Thus, the canonic residues in MHC class I vertebrate molecules are reduced to five. These differences have physical effects in MHC (Major Histocompatibility Complex) class I alpha chain interaction with beta-2-microglobulin but have yet unknown functional effects. Also, introns show specific *Passeriformes* distinction both in size and invariance. The studies reviewed in this paper on MHC structure have been done in wild birds that cover most of the world’s passerine habitats. In this context, we are going to expose the most commonly occurring bird diseases with the caveat that MHC and disease linkage pathogenesis is not resolved. In addition, this field is poorly studied in birds; however, common bird diseases like malaria and Marek’s disease are linked to MHC. On the other hand, the main established function of MHC molecules is presenting microbial and other antigens to T cells in order to start immune responses, and they also may modulate the immune system through NK receptors and other receptors (non-classical class I MHC molecules). Also, structural and polymorphic differences between classical class I molecules and non-classical class I molecules are at present not clear, and their definition is blurred. These passerine exceptional MHC class I molecules may influence linkage to diseases, transplantation, and other MHC presentation and self-protection functions. Further studies in more *Passeriformes* species are ongoing and needed.

## 1. Introduction

Dinosaurs and mammals were thriving on Earth in the Triassic Epoch (300 million years ago; MYA). Both groups survived the Cretaceous extinction about 65 MYA. At present, there are about 5500 mammal species and about 10,000 bird species, and 5700 species of the latter are *Passeriformes* or songbirds [1,2]. The class Aves is considered one of the most evolved forms of dinosaurs [1,2,3,4]. Class I MHC genes and their corresponding proteins in songbirds have been studied and compared to other non-passerine birds and to other available same-vertebrate genes [5]. Only passerines, among birds and other studied vertebrates, conserve Val and Leu amino acids at residues 10 and 96, respectively [5,6]. Also, class I MHC introns of canaries (*Genus Serinus*) and goldfinches (*Genus Carduelis/Spinus*) are studied in this work; they have been extant on Earth since the Miocene Epoch, 21 MYA [6]. *Passeriformes* have been calculated to be on Earth 55 million years ago (Eocene Epoch) [7].

**Figure 1:**
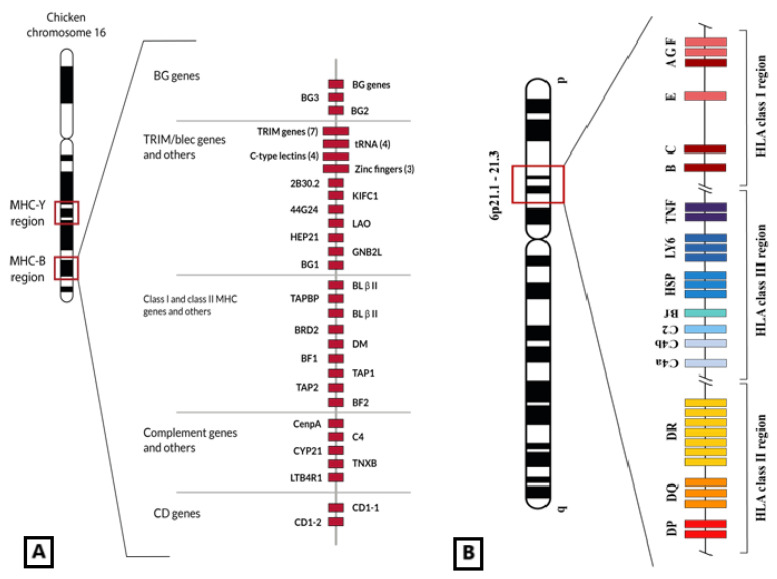
(**A**) Genetic map of the chicken MHC-B region in which MHC-I genes (BF1 and BF2) are included, among others also related to the immune response (C4, TAP, CD1, class II genes or BL, and class II processing genes or DM). BG genes are also close to epithelial and immune cell expression [25]. This set of genes is located in the 16th micro-chromosome of the chicken genome. Figure adapted from [25]. (**B**) Representation of HLA genomic region placed in human chromosome 6 6p21.1–21.3 band. Non-classical class I HLA genes are located in the telomeric part of the HLA region. © by AA-V.

The MHC class I and class II genes are very polymorphic in vertebrates, and their molecules are responsible for presenting peptides to clonotypic receptors on T-cell surfaces to start a specific immune response [8]. MHC class I genes have been widely sequenced and studied in chicken (*Gallus gallus,* order *Galliformes*), wild birds [9,10,11], and mammals [8,12,13,14]. The MHC was first described in chicken [15,16], specifically in the highly related variety Leghorn [12,17]. Chicken MHC-B is clearly different in both structure and function when compared with that of mammals like man (Figure 1); it is simpler and more compact because it has shorter introns, at least in chicken, and its genes are more tightly arranged than in mammals [12,18]. This fact supports the hypothesis that chicken MHC represents a ‘minimum essential MHC’ [12,13] that was also generally attributed to all birds. However, songbird MHC seems to be much more complex than that of chicken because (a) passerine birds have a larger number of genes [19,20]; (b) their introns are longer than those of chicken [5]); (c) pseudogenes have been reported in songbird MHC [19,21], like in humans [22,23]; and (d) passerine genes are highly polymorphic [20,24].

## 2. HLA Class I Region and Molecules

### 2.1. Classic Class I Molecules

**Figure 2:**
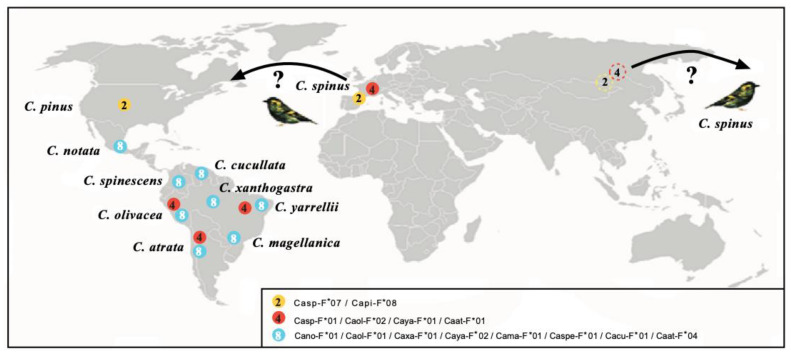
Map showing the geographic location of species sharing MHC-I molecules. Proteins of bird species are depicted with the following names: *Carduelis spinus* (Casp-F*07, FJ266427; Casp-F*01, FJ266399); *Carduelis pinus* (Capi-F*08, FJ266391); *Carduelis notata* (Cano-F*01, DQ257468); *Carduelis spinescens* (Caspe-F*01, DQ257472); *Carduelis olivacea* (Caol-F*02, DQ257471); *Carduelis* atrata (Caat-F*01, FJ266350); *Carduelis magellanica* (Cama-F*01, DQ257467); *Carduelis yarrellii* (Caya-F*01, DQ257475; Caya-F*02, DQ257476); *Carduelis xanthogastra* (Caxa-F*01, DQ257473); and *Carduelis cucullata* (Cacu-F*01, DQ257465). The color of the circles indicates the three different proteins found; the number inside each circle indicates species that share each molecule. GenBank accession numbers are indicated for each allele [79,80,81,82,83]. North Asian dashed circles 3 and 4 mean alleles of *Carduelis spinus*, the probable parental species of North and South American radiations. Question marks mean that *C.spinus* bands have been reported in America outside of its present day range in Europe and Asia. See above indicated references © by AA-V.

HLA (i.e., Human Leukocyte Antigen) class I genes are split up into HLA class I classic genes (HLA-Ia) and class I non-classic genes (HLA-Ib) [26]. HLA-A, -B, and -C (HLA-Ia) were the first described and are expressed in all nucleated cells but mainly in macrophages, dendritic cells, and other immune system cells. HLA-E, -F, and -G (HLA-Ib) codify for very similar proteins to those of classical class I genes (Figure 2), although they differ in some characteristics such as expression location (only certain tissues like cornea or trophoblast), low polymorphism, and immunosuppressive function [27,28]. Structurally, HLA class I molecules are made up of a 270-amino-acid (aac) heavy chain (*α* domains) encoded in the HLA system and a light chain (beta-2-microglobulin; *β*2m) encoded in chromosome 15 [29,30]. *α*1 and *α*2 domains allow the formation of a three-dimensional valve in which the antigen is presented on the surface. *α*3 interacts with the CD8 co-receptor during lymphocyte T immune synapsis [18]. *β*2m has only a structural function.

### 2.2. The Non-Classical Class I HLA Genes: HLA-G, -E, and -F

HLA (Human Leukocyte Antigen) is the human Major Histocompatibility Complex, which is placed in a genomic region containing about 223 genes at chromosome 6p21.3 and encodes for the so-called HLA complex (equivalent to MHC in other species). It has a very important role in the immune response. Genes named classical class I genes for historical reasons (HLA-A, HLA-B, and HLA-C) encode for molecules whose function is presenting antigen peptides to clonotypic T-cell receptors placed on the surface of CD8+ cells; on the other hand, the non-classical class I molecules (HLA-G, HLA-E, and HLA-F) (Figure 1) are mainly related to control functions of the immune system cells [31,32,33]. The HLA-G function was assigned to be an immune control molecule, firstly found at the maternal–fetal interface and later in many other tissues, but its function was thought to be only inducing maternal–fetal tolerance [32,34,35,36]. Dan Geraghty et al. [37] named this protein HLA-6.0, the new gene that they discovered. The structure of the HLA-6.0 protein was similar to HLA-A, -B, and -C class I molecules, but a stop codon inhibited the translation of a large part of the cytoplasmatic region in the HLA-6.0 molecule. Regarding the promoter region of the *HLA-6.0* gene, it was almost identical to that of the MHC-Qa mouse gene; in addition, both of these genes had equivalent substitutions, deletions, and other changes in their DNA sequences [37]. It was proposed [38] that MHC-Qa was a mouse HLA-G homologous with a similar gene and protein structure; MHC-*Qa* also has soluble isoforms similar to HLA-G5, G6, and G7 isoforms in man (Figure 1). In addition, it is proved that *Qa-1b* (MHC-Qa non-classical class I gene in mouse) is homologous to HLA-E (see HLA-E “Evolution” section). The full HLA-G protein has an extracellular structure almost identical to that of the classical HLA molecules, though its main function is not peptidic antigen presentation. Also, HLA-G hinders the cytotoxicity of T CD8+ and NK cells by interaction with leukocyte receptors, like LILRB1 (LIR1/ILT2), LILRB2 (ILT4), and KIR2DL4 (CD158d) [33,39,40,41,42,43,44].

However, the HLA-G gene and protein expression patterns are different in certain ways from those of classical HLA class I proteins, as follows: (a) It has a more restricted tissue expression in normal metabolic circumstances [45]. It has been found to be expressed on the maternal–fetal interface, particularly in the cellular line of extravillous cytotrophoblast [6], cornea, proximal nail matrix, thymus, hematopoietic stem cells, and pancreas [46,47,48,49,50,51]. HLA classical class I molecules are widely expressed in all body tissues. Non-classical class I HLA molecules are more restricted regarding tissue localization, and their function is different, as they are not antigen-presenting molecules [33]. The variation of presented antigens compared with those of classical class I MHC proteins is very reduced, possibly because of their lower polymorphism [52]. These non-classical class I proteins also modulate immune function through TCR-independent interactions (see below). (b) It has several membrane-bound and also soluble isoforms because there is an alternative splicing of the complete mRNA of HLA-G [32,33]. (c) A short cytoplasmic tail is found because there is a stop codon at exon 6 [32,33]. (d) At the moment, a relatively low HLA-G molecule polymorphism has been found, although it is quickly growing (Figure 2) [32,33,53]. (e) It has a peculiar 5′UTR (5′ upstream regulatory region), which differs from HLA classical class I genes [54,55]. (f) The 5′ promoter region [32,56,57,58,59,60] and the 3′UTR (3′ untranslated region) present some polymorphisms that are specifically linked to susceptibility to certain diseases [61]. However, it has been found that HLA-G presents endogenous peptides at the surface cells of the placenta trophoblast [62]; this does not occur with other HLA classical class I molecules, which may also be expressed on the same cell surface [63], except for HLA-C [64]. Therefore, HLA-G is extant on the outer placenta cytotrophoblast cell line of fetal and contacts,: (a) with maternal blood NK cells through their killer immunoglobulin-like receptors (KIRs), both inhibitory and activating receptors; (b) the so-called white blood cell LIRs (leukocyte immunoglobulin-like receptors); and (c) the complex of CD94-NKG2 receptors. This variety of HLA-G interactions results in achieving maternal tolerance to the fetus and a lack of rejection [43]. Also, HLA-G interacts with both cytolytic and regulatory [65,66] T cells through their respective surface clonotypic T-cell receptors [67]. On the other hand, the HLA-E molecule on the surface of cytotrophoblast placental cell polymorphisms sends signals to the mother’s immune system to enhance fetal tolerance: a complex of HLA-E allelic protein bound to peptides that come from leader peptides of class I HLA molecules. These HLA-E/peptide complexes interact with maternal CD94-NKG2 and clonotypic T-cell receptors [68,69].

In contrast with HLA-E and HLA-G physiology, HLA-F has not been so widely studied and has apparently deserved less attention. However, it is known that HLA-F also promotes fetal normal growth [70], and, in addition, this molecule modulates the immune system of the peripheral nervous system; it has been found in amyotrophic lateral sclerosis that HLA-F hinders motor neuron death by its binding to inhibitory KIR3DL2 receptors [71]. Also, HLA-F enhances the antiviral response to HIV (human immune deficiency virus-1) by binding to activating KIR3DS1 receptors of NK cells [72]. This is a very important HLA-F immune regulatory function because its binding to KIR3DS1 receptors has been found to be crucial in other diseases in which KIR3DS1 shows a pathogenetic function [73]. In summary, control or modulation by the HLA-F protein has been found to be critical in the pathogenesis of some diseases. Notwithstanding, HLA-F’s tertiary properties and structure need to be further studied in order to establish a relationship with its pathogenetic activity in diseases. Computational predictions conclude that HLA-F has only a partial open groove and normal MHC folding [67,74].

## 3. MHC Trans-Species Evolution

Mammals (apes) and birds have been shown to present MHC trans-species evolution [75,76]. This usually happens when speciation has rapidly occurred while MHC genes still remain. This fact is usually explained by MHC molecules’ adaptation to a specific environment with specific pathogens to work with, and evolutive forces for the invariance are strong enough to resist speciation timing. This trans-species evolution of MHC genes could also explain why fewer alleles are found in wild species than in “artificial“ (or more inbred) ones, like human, mouse, and domestic chicken [76,77]. Human, laboratory mouse, and domestic chicken are considered “artificial” models because they have undergone a bottleneck and subsequent inbreeding. This parental relatedness and relative chromatide similarities facilitate crossover at meiosis and, therefore, would lead to excessive MHC allelism since this has been shown to be the main MHC mechanism through which it generates diversity in this system—not through point mutations [76,77,78]. MHC class I DNA sequences of Passerine birds studied by us [79] clearly showed trans-species evolution: two DNA matches exist between MHC-I alleles of different South American siskin species. In addition, the Eurasian siskin shares an identical MHC protein allele with the pine siskin (Central North America radiation). The Eurasian siskin also shows a protein allele shared with three different South American siskin species. More protein allele sharing has been identified with one single MHC protein in eight South American siskin species, including the extant parental species *C. notata* (Figure 2). All MHC genes transmit the corresponding MHC allelic proteins to the descendant species if they have time (and no selective forces exist) before speciation occurs. South American siskins separated from the North American siskin radiation about 5-4 million years ago, and this study is detailed in Figure 2 [80]. The closest mt DNA genetic relationship occurs between the Eurasian siskin and the pine siskin [80,81,82,83]. This is further supported because these two species share one MHC class I protein allele. Apparently, the Eurasian siskin (or an extinct relative) was thriving around America, Asia, and Europe and has apparently given both North American and South American siskin radiations directly or through the North American radiation [80,81,82,83]. In summary, our own data with mitochondrial mt cyt b, together with the first described evidence of trans-species MHC evolution in birds, lead us to postulate that the Eurasian siskin is the extant ancestor of present-day North and South American siskins/goldfinches *Carduelis* species [80,81,82,83].

## 4. Songbirds Have at α1 Chain Val10 and Not the Canonic Vertebrates Thr10 Amino Acid and in α2 Chain Leu96 and Not the Canonic Vertebrates Gln96 Residue 

**Figure 3:**
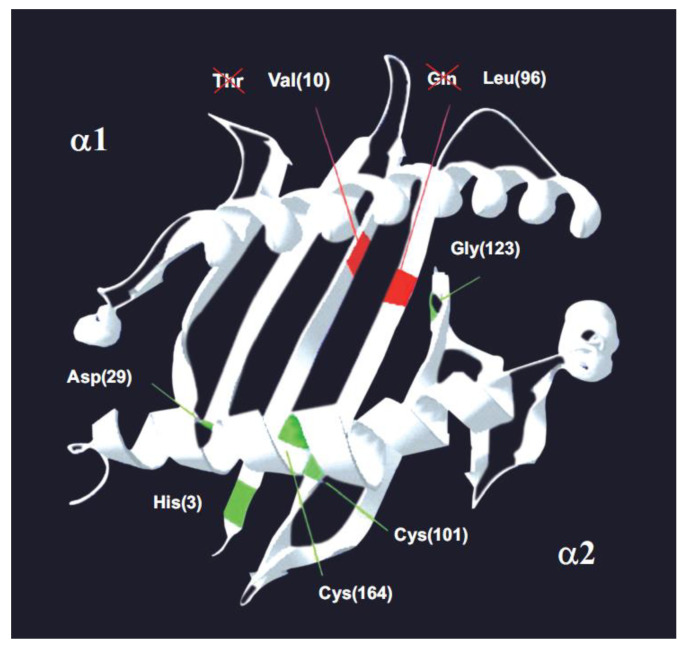
The MHC-I molecule. A model of α-1 and α-2 domains on a songbird class I histocompatibility molecule. Green lines: the five positions conserved in vertebrates [84], including chicken and pheasant. Red lines: the two conserved positions (10 and 96) that have an amino acid change in songbirds–passerines compared to other vertebrates. *Passerines* bear Val10 and Leu96. In brackets: amino acid position number [5]. The 7 invariant or canonic amino acid positions in MHC class I molecules in vertebrates are shown in *Passeriforme* birds, where Val 10 and Gln 96 are observed instead of Thr 10 and Leu 96. The conclusion is that there are not seven conserved positions in vertebrates, but five conserved positions because, at the least, passerines have lost two out of the seven canonic positions. © by AA-V.

Table 1 shows all vertebrates and bird species which are tested in this study and comprise most of Earth’s continents and thrive in various habitats. MHC class I molecules have been found to preserve seven canonical amino acid positions in vertebrates since the appearance of jawed fishes on Earth to humans (from Devonic Epoch, 300 MYA, until present) [83,84]. These conserved amino acids are Thr10, Asp29, and Asn86 in the α1 chain; and Gln96, Gly100, Cys101, and Cys164 in the α2 chain. The α1 and α2 protein domains form valve-like structures; antigenic molecules are accommodated inside this valve and are presented to the specific T-lymphocyte receptor to begin an immune response [8,85]. These conserved canonical residues must be under powerful evolutive forces to help keep the class I MHC valve-like structure. However, two of these permanent residues at such a wide time-evolutionary scale are NOT present in songbirds (*Passeriformes*), which account for about half (five thousand) of the extant birds: Val10 has been substituted for Thr10 on the α1 chain, and Leu96 has been substituted for Gln96 on the α2 chain (Figure 3; Table 2 and Table 3). Our research found these results in our MHC studies in wild passerine birds studied so far, including warbler and zebra finch predicted sequences [86].

**Table 1:** Vertebrate English and Latin names of the species studied (including wild songbirds), and GenBank accession numbers are detailed. -: gap or unknown. *: predicted sequence. *Passeriformes*, other birds, primates, other mammals, reptiles, fishes, and wild passerines are thriving in an area that comprises all of Earth’s continents and sea ranges [43,44]. It is noteworthy that jawed fish (like zebra fish or carp) first had their appearance on Earth at least 400 MYA (see Ref. [3] from Table 1 at Ref. [5]).

English Name	Name	GenBank No.	Position 10	Position 96
Zebra fish	*Danio rerio*	AAF20179	-	Gln
Carp	*Cyprinus carpio*	[5]	Thr	Gln
African clawed frog	*Xenopus laevis*	[5]	Thr	Gln
Snake	*Nerodiasipedon*	[5]	-	-
Ameiva lizard	*Ameiva ameiva*	[5]	Thr	Gln
Cow	*Bos taurus*	ABW70136	Thr	Gln
Dog	*Canis familiaris*	NP_001014767	Thr	Gln
Horse	*Equus caballus*	NP_001075976	Thr	Gln
Mouse	*Mus musculus*	AAY85367	Thr	Gln
Sheep	*Ovis aries*	CAJ57269	Thr	Gln
Rat	*Rattus norvegicus*	CAA74333	Thr	Gln
Greater horseshoe bat	*Rhinolophu sferrumequinum*	ACC68844 *	Thr	Gln
Pig	*Sus scrofa*	ACA33862	Thr	Gln
Short-beaked echidna	*Tachyglossus aculeatus*	AAM54212	-	Gln
Commongibbon	*Hylobates lar*	AAB08074	Thr	Gln
Orangutan	*Pongo pygmaeus*	AAK67485	Thr	Gln
Western gorilla	*Gorilla gorilla*	CAA43100	Thr	Gln
Chimpanzee	*Pan troglodytes*	BAC78189	Thr	Gln
Bonobo	*Pan paniscus*	AAY59433	Thr	Gln
Human (HLA-A2)	*Homo sapiens (HLA-A2)*	BAA07530	Thr	Gln
Human (HLA-B)	*Homo sapiens (HLA-B)*	CAA06616	Thr	Gln
Human (HLA-C)	*Homo sapiens (HLA-C)*	CAB02408	Thr	Gln
Chicken	*Gallus gallus*	AY489160	Thr	Gln
Japanese quail	*Coturnix japonica*	D29813	Thr	Gln
Great reed warbler	*Acrocephalus arundinaceus*	CAA06566	Val	Leu
Black siskin	*Carduelis atrata*	DQ257462	Val	Leu
Black-capped siskin	*Carduelis atriceps*	FJ268821	Val	Leu
European goldfinch	*Carduelis carduelis*	FJ266447	Val	Leu
Citrilfinch	*Carduelis citrinella*	DQ257482	Val	Leu
Lawrence’s goldfinch	*Carduelis lawrencei*	FJ314425	Val	Leu
Pine siskin	*Carduelis pinus*	FJ266376	Val	Leu
Eurasian siskin	*Carduelis spinus*	FJ266399	Val	Leu
Common rosefinch	*Carpodacus erythrinus*	ACL31612.1	-	Leu
Chaffinch	*Fringilla coelebs*	DQ257477	Val	Leu
Yellow-rumped seedeater	*Serinus atrogularis*	DQ257479	Val	Leu
Africancitril	*Serinus citrinelloides*	DQ257484	Val	Leu
Lemon-breasted seedeater	*Serinus citrinipectus*	DQ257483	Val	Leu
White-bellied canary	*Serinus dorsostriatus*	DQ257486	Val	Leu
Yellow canary	*Serinus flaviventris*	DQ257487	Val	Leu
Streaky-headed seedeater	*Serinus gularis*	DQ257489	Val	Leu
Yellow-fronted canary	*Serinus mozambicus*	DQ257491	Val	Leu
Streaky seedeater	*Serinus striolatus*	DQ257493	Val	Leu
Tibetan serin	*Serinus thibetanus*	DQ257496	Val	Leu
Zebra finch	*Taeniopygia guttata*	LOC100231469 *	Val	Leu

In summary, songbirds have two different out of the seven canonic MHC class I amino acid residues that are present in vertebrates and also in other birds with more terrestrial thriving places, like chicken (*G. gallus*) and quail (*Coturnix japonica*) (Table 1 and Table 2). Sibley and Alquist offered a DNA classification not based on sequencing but on DNA RFLPs [3]. We have demonstrated that *Passeriformes* (accounting for 4600 species) of *Serinus* and *Carduelis Genera* have two residue changes in contrast with other bird species like chicken and quail (Table 1). Interactions of the two different songbird amino acid residues in tri-dimensional MHC proteins consist of the following:

1. The amino acid residues Thr10 and Gln96 side chains that are canonic in all vertebrate MHC class I proteins do not present any interaction with each other or with the presented peptide within the molecular MHC class I valve.
2. The side chains of these two exceptional songbird residues interact with the β2-microglobulin (B2M) molecule, and they provide security to maintain the tertiary structure of the complete molecule.
3. In vertebrates other than birds (except chicken and quail), the canonic residues show these chemical peculiarities:
  - B2M molecules have two hydrogen links to the Gln96 residue of the MHC class I chain: one link is to His31, and the second one is to the Trp60 amino acid of the B2M molecule.
  - The Thr10 amino acid of the MHC chain is kept between residues Met54 and Phe62 of the B2M molecule; also, one H_2_O molecule is stuck to the Thr10 residue of the MHC peptide chain.

Table 1 and Table 2 and Figure 3 show which songbird species have these two canonic amino acid changes or exceptions and where the changes are in the MHC molecule. The effects of these changes are the following:

  - Leu96 substitutes for Gln96 in the MHC chain, and this results in two hydrogen bonds to the B2M molecule disappearing, affecting the complete molecule stability. Also, the class I molecules of other *Passeriformes*, like *Taeniopygia guttata* (zebra finch), *Acrocephalus arundinaceus* (great reed warbler), and common rosefinch, have also been analyzed from the results in [38,87,88].
  - The Val10 change in passerine birds from the canonical vertebrate Thr10 may counteract the effects of residue 96 change with regard to the B2M attachment affinity to the MHC class I α chain and the binding stability of the two molecules. This is due to the fact that van der Waals interactions with B2M appear at the level of residues Met54 and Phe62; in addition, the H_2_O molecule, which was trapped with the canonical vertebrate residue, is now released with an entropy change, i.e., an entropy gain.

**Table 2:** MHC class I amino acid residue differences in sequences. Residue 10 in alpha-1 chain amino acid sequences of species MHC molecule. Residue 96 in MHC alpha-2 chain amino acid sequences of species. Passerine birds show exceptional changes on these two residues in contrast with other vertebrates. Passerines show Val10 instead of canonical Thr10 in vertebrates, which is replaced in the alpha-1 chain of MHC molecules, and they have Leu96 replacing canonical Leu96. This has been tested in passerine species thriving in a range that comprises most of the world. Non-passerine birds and other vertebrates show Thr and Gln at positions 10 and 96, respectively, which are found in non-passerine birds and all other analyzed vertebrates. The canonic vertebrate positions appeared in Earth vertebrates approximately 300 MYA, while passerines or songbirds appeared on Earth about 35 MYA in the Middle Eocene Epoch (see Figure 4, although some authors think that the passerine appearance on Earth was 30 MY before); thus, these are two exceptions out of the seven canonic residues in MHC class I molecules. The numbering of 10 and 96 residue positions is referred to as the human HLA-A2 molecule model [84,85,89]. The zebra finch (*Taenopygia guttata*) sequence is taken from [86]. The common rosefinch α2 domain sequence is referenced in [5,88]. The geographic origin of wild bird samples is detailed in Table 1 [79].

Model	10	96
HLA-A2	Thr	Gln
***Danio_rerio***	-	-
***Xenopus_laevis***	-	-
***Ameiva_ameiva***	-	-
***Bos_taurus***	-	-
***Canis_familiaris***	-	-
***Mus_musculus***	-	-
***Sus_scrofa***	-	-
***Pan_troglodytes***	-	-
***Coturnix_japonica***	-	-
***Gallus_gallus***	-	-
***Acrocephalus_arundinaceus***	Val	Leu
***Carduelis_atrata***	Val	Leu
***Carduelis_carduelis***	Val	Leu
***Carduelis_lawrencei***	Val	Leu
***Carduelis_pinus***	Val	Leu
***Carduelis_spinus***	Val	Leu
***Fringilla_coelebs***	Val	Leu
***Serinus_atrogularis***	Val	Leu
***Serinus_thibetanus***	Val	Leu
***Taeniopygia_guttata***	Val	Leu

**Figure 4:**
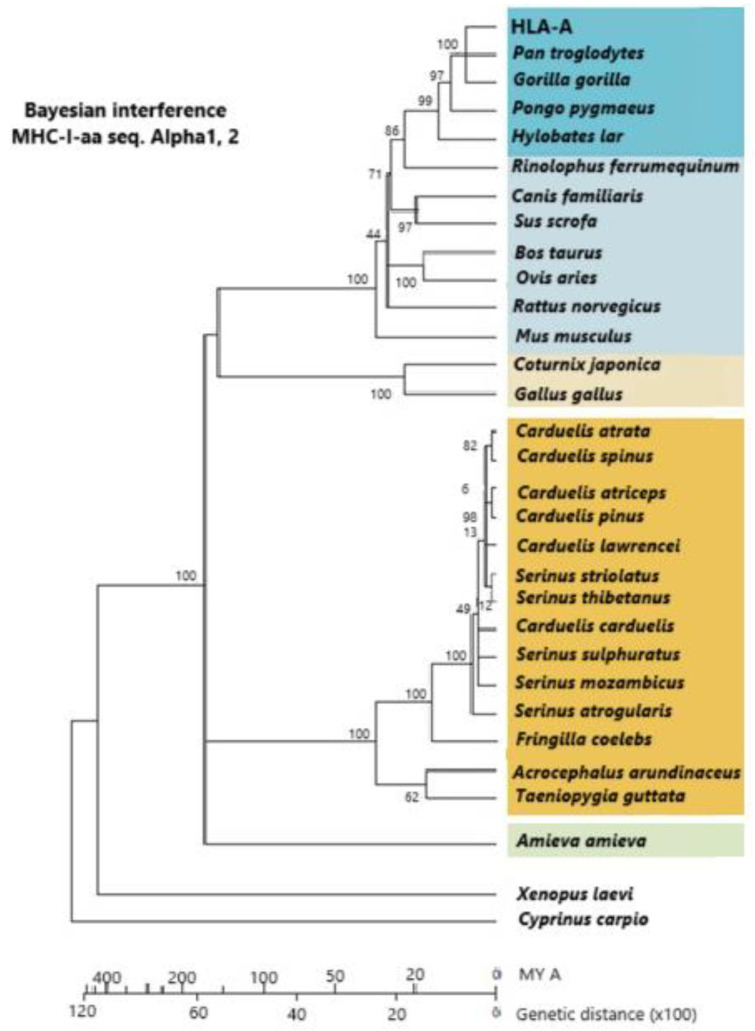
A phylogram constructed with a linearized Bayesian inference methodology. It is built from alpha-1 and alpha-2 amino acid sequences of MHC class I molecules. Dark orange: *Passeriformes*; light orange: other birds; dark blue: primates; light blue: other mammals; green: Amieva lizard; white: other vertebrates. Vertebrates group together within groupal subdivisions. The vertebrate groupage is reptiles, mammals, more “terrestrial” birds, and more “aerial” birds. The use of molecules more reliable than MHCs (which are under selection pressure) is preferably carried out for calculating lineage time divergence (i.e., mt cyt b DNA molecules, which are subjected to a constant evolutive pressure). Passerine appearance on Earth is 35 MYA at the Middle Eocene Epoch, although some authors think that this date should be put back about 30 MY before. [5,79] © by AA-V.

## 5. Relatedness of MHC Class I Molecules Among Passeriformes Birds and Other Birds and Vertebrates

Figure 4 shows how Passeriformes are closely related among themselves and separate from other birds (chicken and quail). The alpha-1 and alpha-2 domains of class I MHC molecules are used to construct the phylogenetic dendrogram. Since linearized Bayesian inference permits an approximate calculation of time (Figure 4, bottom line), it is also observed that *Galliformes* are older on Earth than *Passeriformes* [79]. It may be taken into account that half of the extant bird species are *Passeriformes* or songbirds, in contrast to all other extant birds, which are classified into 12 different species. *Passeriformes* are smaller and more aerial, and this may have helped them to pass more easily the 65 MYA dinosaur/bird extinction bottleneck, as the mainstream thinking is that birds and dinosaurs are closely related or that birds are extant surviving dinosaur lineages [79]. On the other hand, MHC class I introns of *Passeriformes* are substantially different from those of *Galliformes* (Arnaiz-Villena et al. unpublished, [79]; see Section 6).

## 6. Intron 2 of MHC Class I Molecules in *Passeriformes*

Wild *Passeriformes* were used throughout the studies presented in this work. Species were chosen whose living range and origin (Table 1) cover all world ranges. Table 3 shows that the geographically and genetically closest species, according to mitochondrial DNA analyses, have the biggest similarity percentage regarding similarity in nucleotide positions (Arnaiz-Villena et al., unpublished). Also, *Passeriformes* introns are larger than expected (in contrast to chicken smaller size): intron size is similar to that of human MHC class I intron 2. It was observed that 207 nucleotide positions out of 312 were conserved in the studied *Passeriformes* during approximately 15 million years (Figure 4, Table 3).

**Table 3:** Similarity percentage between species studied by using nucleotide base positions of MHC class I intron 2. (79) © by AA-V.

	1	2	3	4	5	6	7	8	9	10	11	12	13	14	15	16
***1. Carduelisatrata***																
***2. Carduelisatriceps***	97.7															
***3. Carduelis carduelis***	97.1	97.4														
***4. C.ardueli scitrinella***	96.4	96.7	95.5													
***5. Carduelis pinus***	98.1	99.7	97.7	97.1												
***6. Carduelis spinus***	98.7	97.7	97.1	96.5	98.1											
***7. Serinus atrogularis***	97.1	97.0	96.1	95.5	97.1	97.1										
***8. Serinus citrinelloides***	97.4	97.1	97.1	95.8	97.4	97.4	98.4									
***9. Serinus citrinipectus***	96.8	96.5	95.8	95.8	96.8	96.8	97.1	97.4								
***10. Serinus dorsostriatus***	97.4	97.1	96.4	95.8	97.4	97.4	97.7	98.1	97.4							
***11. Serinus flaviventris***	96.4	96.1	95.5	94.8	96.4	96.5	97.4	97.7	96.5	97.1						
***12. Serinus gularis***	98.1	97.7	97.1	96.4	98.1	98.1	99.0	99.4	98.1	98.7	98.4					
***13. Serinus mozambicus***	96.8	97.1	96.4	95.8	97.4	96.8	97.7	98.1	98.7	97.4	97,1	98.7				
***14. Serinus striolatus***	97.4	97.1	97.1	95.8	97.4	97.4	97.7	98.7	96.8	97.4	97.1	98.7	97.4			
***15. Serinus thibetanus***	98.4	97.4	96.8	96.1	97.7	97.7	96.8	97.1	96.5	97.1	96.1	97.7	96.4	97.1		
***16. Fringilla coelebs***	84.5	84.5	84.2	83.5	84.5	84.2	83.5	84.5	83.9	84.2	82.4	84.5	83.9	83.5	83.5	

## 7. MHC and Bird Diseases

MHC genes may also influence susceptibility or resistance to certain diseases. The research field on this topic in birds is yet undeveloped compared to studies in humans and mice. However, there are some bird diseases that have been described to be associated with MHC that are going to be briefly referred to in this section (see Table 3).

### 7.1. Marek’s Disease (MD)

Marek’s disease is a frequent disease in grange-bred chickens. It is caused by an oncogenic herpesvirus that causes serious immunosuppressive diseases, lymphoma, and other malignant diseases [90]. It is proved that chickens with resistance to MD have a specific size-determined (1.5 Kb) MHC class I transcript and no other [91]. The vaccine for MD is sometimes useless in certain breeding granges due to unknown reasons [90].

### 7.2. Malaria

MHC alleles or a group of neighbouring MHC genes/alleles, as measured by RFLP or other methodologies (haplotypes), have been found to be linked to malaria bird disease. Disease severity and prevalence have been associated with certain MHC-I alleles in *Cyanistes caeruleus* [92], *Achricephalus arundinaceous* [93], *Geothlypistrichas* [11], and *Melospizamelodea* [94]. The prevalence and severity of malaria in *Parus major* [95] and *Achrocephalusschoenobacnus* [96] are associated with MHC haplotypes. The prevalence of malaria has also been associated with MHC in *Ficedulaalbicollis* [97].

### 7.3. Bacterial Skeletal Disease

Chicken skeletal lesions, bacterial pathogens, and MHC genotype are apparently involved in the observed clinical signs. A4 and A12 MHC class I haplotypes are associated with this pathology. The disease causes lameness due to tenosynovitis, arthritis, and femoral and tibiotarsal osteomyelitis, and it is of significant importance in chicken breeding. Many times, it has been found to be associated with *Staphylococcus aureus*, but it also may be caused by other *Staphyloccocus* agents, such as *S. agnetis*, *S. cohnii*, *S. epidermidis*, *S. hyicus*, or *S. simulans* [98].

### 7.4. Avian Pox

Avian pox is a viral disease that affects wild and domestic birds. It is caused by the avipoxvirus, which is a member of the *Poxviridae* family. The disease is characterized by the formation of wart-like lesions on the skin, beak, and feet of affected birds. Avian pox is a common disease that affects a wide range of bird species, including songbirds, raptors, waterfowl, and game birds. In this article, we will explore the causes, impacts, and management of avian pox in wildlife populations. Susceptibility is also related to MHC [99].

### 7.5. Ectoparasite Infection

Four congenic lines of chickens, differing only at the MHC, were comparatively infested with a cosmopolitan ectoparasite of birds—Northern Fowl Mite (NFM)—which is also a serious pest species of poultry. Mite infestations were monitored over time, and mite densities (weekly and maximum) were compared among lines. Chickens with the MHC haplotype B21 were relatively resistant to NFM compared with birds in the B15 congenic line [96,100,101]. The NFM infestation produces a huge energetic cost in the individual due to a defective immune response causing less egg weight and other pathologies [102].

### 7.6. Infectious Bronchitis (IBV)

To study disease resistance in chickens, MHC congenic chicken lines that share the same genetic background with differences exclusively in their MHC B locus have been developed [103,104,105,106]. Using these chicken lines as animal models, associations between MHC haplotypes and disease resistance or susceptibility have been described for several infectious pathogens, including *Coccidia* [107,108], pathogenic bacteria [109,110,111,112], oncogenic viruses [113,114,115,116,117,118,119,120], and other viruses, including IBV [121,122,123,124,125,126,127,128,129,130]. The disease causes respiratory deficiency, leading to weight and egg production decreases [131].

Recent advances in MHC avian immunology and several infectious diseases are noteworthy: particular epitopes binding B2 haplotype chickens that confer resistance to infectious avian diseases have been identified [132]. Also, some of the CD8+ T responsive cell epitopes have also been discovered, and this may be crucial for effective vaccine development [133]. In addition, CD8+ T-cell-specific B haplotypes in duck and twelve CD8+ influenza virus T-cell epitopes have been reported in a progressive way to reach a fully useful vaccine [134].

## 8. Conclusions

1. The MHC class I molecules analyzed in songbirds or *Passeriformes* have only 5 out of 7 exceptional canonic or invariant residues (Figure 3): *Passeriformes* Val10 and Gln92 differ. Whether the tested MHC class I molecules are classical or non-classical is not known since the structural difference between both kinds of molecules is now blurred [135]. These two residues change the concept that MHC class I molecules have seven invariant canonic residues in vertebrates, as the canonic changes are only five when counting passerine birds.

2. These exceptional two residues have an effect on the MHC class I alpha chain joining to beta-2-microglobulin, whose entropic and physical interaction consequences are explained in the text, but no functional consequences may yet be inferred (Table 1 and Table 3). More studies are ongoing, and it is necessary that more species be searched.

3. Also, *Passeriformes* birds’ MHC class I intron 2 shows remarkable preservation over time; it has been quite stable for millions of years. All these studies have been performed with wild birds DNA (Figure 2, Table 1).

4. The linkage of MHC to diseases has been scanty in birds in comparison to the too many done (without a pathogenesis result) in man and mouse models. However, some microbes and parasites are associated with MHC in birds, like malaria, Marek’s disease, and mites. The susceptibility to these and other diseases has been related to both specific MHC alleles and MHC gene duplication.
